# Family medicine vocational training and career satisfaction in Hong Kong

**DOI:** 10.1186/s12875-019-1030-8

**Published:** 2019-10-20

**Authors:** K. P. Lee, C. Wong, D. Chan, K. Kung, L. Luk, M. C. S. Wong, D. Chao, V. Leung, C. W. Chan, W. Ko, T. F. Leung, Y. H. Chan, H. T. Fung, M. K. Lee, S. Y. S. Wong

**Affiliations:** 1 JC School of Public Health and Primary Care, The Chinese University of Hong Kong, Sha Tin, Hong Kong; 2 Hospital Authority, Hospital Authority Building, 147B Argyle Street, Kowloon, Hong Kong

**Keywords:** Doctors’ satisfaction level, Vocational training, Family medicine training, Career satisfaction, Job satisfaction

## Abstract

**Background:**

Postgraduate vocational training in family medicine (FM) is essential for physicians to build capacity and develop quality primary care. Inadequate standards in training and curriculum development can contribute to poor recruitment and retention of doctors in primary care. This study aimed to investigate: 1) the satisfaction level of doctors regarding vocational training in family medicine and associated demographics; and 2) the satisfaction level of doctors regarding their family medicine career and associated factors.

**Method:**

This is a cross sectional study of all family medicine physicians across all government-funded primary care clinics (GOPCs). The study questionnaire consisted of items from a standardized and validated physician survey named the Physician Worklife Survey (PWS) (Konrad et al., Med Care, 1999). We selected three scales (7 items) relating to global job satisfaction, global career satisfaction and global specialty (family medicine) satisfaction with additional items on training and demographics. All significant variables in bivariate analyses were further examined using stepwise logistic regression.

**Results:**

Out of 424 eligible family medicine physicians, 368 physicians successfully completed the questionnaire. The response rate was 86.8%. Most participants were male (52.6%), were aged between 35 and 44 years (55.5%), were FM specialists (42.4%), graduated locally (86.2%), and had postgraduate qualifications. Eighty-two percent (82%) of participants were satisfied with their training. Having autonomy and protected time for training were associated with satisfaction with FM training. Satisfaction with family medicine as a career was correlated with physicians’ satisfaction with their current job. Doctors who did not enroll in training (*p* < 0.001) and physicians who were older (*p* = 0.023) were significantly less satisfied. Stepwise multivariate regression showed that doctors who subjectively believed their training as “broad and in depth’ had higher career satisfaction (*p* < 0.001).

**Conclusion:**

Overall, the satisfaction level of physicians on current family medicine training in Hong Kong was high. Having autonomy and protected time for training is associated with higher training satisfaction levels. Perceiving FM training as “broad and in-depth” is associated with higher family medicine career satisfaction.

## Background

Postgraduate training in family medicine (FM) is essential for physicians to build their capacity and develop quality primary care. Inadequate standards in training and curriculum development may contribute to poor recruitment and retention of doctors in primary care [1]. In Hong Kong, students undertake an undergraduate medical degree as their first degree. Postgraduate family medicine training is provided by the Hong Kong College of Family Physician (HKCFP). The training of family medicine specialists in Hong Kong consists of 4 years of ‘basic training’ (2 years in hospital and 2 years in family medicine clinics in the community) and 2 years of ‘higher training’ in family medicine clinics (when trainees are required to conduct research or an audit, and learn clinic management and administration). Trainees receiving ‘basic training’ and ‘higher training’ are called ‘basic trainees’ and ‘higher trainee’ respectively. To obtain eligibility to enroll into higher training, basic trainees must pass the conjoint examinations organized by HKCFP and the Royal Australian College of General Practitioners (RACGP). Doctors with more than 5 years of experience in general practice can still apply for higher training after passing the co-joint examinations, and are not required to complete basic training [2]. Training is conducted primarily during training posts in general out-patient clinics (GOPCs), which are Government-funded clinics managed by the Hospital Authority (HA). Meanwhile, trainers are family medicine specialists appointed by the HKCFP [2]. Other doctors working in GOPCs include trained family medicine specialists and ‘service doctors’, who do not undergo formal vocational family medicine training.

In recent years, there has been increasing guidance for graduate medical education in family medicine which emphasizes training in terms of resources, programs and evaluation [3]. Meanwhile, family medicine career satisfaction has been associated with residency education that is broad and in depth [4]. The duration of postgraduate vocational FM training in Hong Kong is 6 years, which is longer than most programs where the specialty of family medicine is advanced, e.g., 2–4 years training in Canada [5], US [6], UK [7] and Australia [8]. A qualitative study of vocational FM training in Hong Kong in 2010 revealed high variability in training standards and content, with limited training opportunities and heavy clinic workload [9]. A recent addition to the vocational program requires trainees to do research during their training, which is also present in other overseas training programs [10, 11]. Almost all FM training is conducted in the public sector in GOPCs, with only a couple of trainees in FM training in private sector each year. The objectives of this study are to investigate: 1) the satisfaction level of doctors regarding vocational training in family medicine and associated demographics; and 2) the satisfaction level of doctors regarding their family medicine career and associated factors. This study will survey doctors working in the public sector in Hong Kong.

## Method

This is a cross sectional study of family medicine physicians across all 73 GOPCs, which were divided into seven ‘clusters’ governed by HA. These clusters were pre-defined by the HA for resources allocation reasons; each cluster has an in-charge doctor to co-ordinate FM training. Questionnaires were sent to all doctors working in the GOPCs from May 2015 to January 2016. The questionnaires were delivered to training coordinators of the seven clusters of HA, and distributed by internal mail to individual doctors. All physicians were informed that the questionnaire would be anonymous and were instructed to return completed questionnaires directly to the study team (The Chinese University of Hong Kong) either by fax or through internal mail. An e-mail reminder was sent to all GOPCs doctors around 1–2 months after the first batch of questionnaires were sent out. Because it took time for questionnaires to be distributed and returned by internal mail, a second round of questionnaires was sent after 6 months. Physicians were encouraged to fill in the second questionnaire if they had not participated in the first round. Those eligible to participate included basic trainees, higher trainees, FM specialists, and service doctors as previously defined.

### Instrument – family medicine training satisfaction questionnaire

The study questionnaire consisted of items from a standardized and validated physician survey named the Physician Worklife Survey (PWS) [12]. We selected three scales (7 items) relating to global job satisfaction, global career satisfaction and global specialty (family medicine) satisfaction (Additional file 2: Appendix 1). Additional items were based on questions by Young et al. [4] which were selected and adapted by the study team of family medicine physicians and academics. A pilot study of 10 family medicine trainees was conducted to ensure face and content validity. Responses were set at a 4-point Likert scale (strongly disagree, disagree, agree and strongly agree) (Additional file 2: Appendix 2). In addition, demographic information including sex, age, working location, place of graduation, postgraduate qualifications, trainee status, sessions of training in the past 12 months, and training modality were collected. Participants were also asked about the likelihood that they would recommend the training to others (on 10-point Likert scale).

### Data analysis

Continuous variables were presented as mean ± standard deviation and categorical variables as count and percentage. Two sample independent t tests, ANOVA or Kruskal Wallis test were used for continuous variables, while chi-squared tests or Fisher Exact test for categorical variables. Physicians who responded with ‘agree’ or ‘strongly agree’ to the question ‘I am satisfied with the training provided in GOPC setting over the last 12 months’ were categorized as satisfied with the training and the proportion of doctors satisfied with training was the primary outcome. Those who were not satisfied with training were compared in terms of demographics and educational characteristics. Frequencies and descriptive statistics were computed on survey items. To investigate the independent factors of satisfaction on the training, all significant variables in the bivariate analyses with *p*-value of < 0.1 were further examined using stepwise logistic regression.

The secondary outcome variable was the sum of Likert scores from two questions on the PWS for family medicine career satisfaction: ‘If I were to choose again, I would choose to be a family physician’ and ‘I would recommend family medicine to a student seeking advice’. Spearman correlation coefficients were used to evaluate bivariate relationships between Family Medicine Career Satisfaction Score and other continuous variables. The independent factors of the score were quantified using linear stepwise regression analysis.

Statistical analyses were performed using the statistical package SPSS version 21 (SPSS Inc., Illinois, US). Two-tailed *p*-values at level of 0.05 or less were considered to be statistical significance. 95% Confidence intervals were provided wherever appropriate.

## Result

Out of 424 eligible family medicine physicians, 368 successfully completed the questionnaire. Thus, the response rate was 86.8%.

### Respondent characteristics

Most participants were male (52.6%), aged between 35 and 44 years (55.5%), FM specialists (46.8%), graduated locally (86.2%) and had different levels of postgraduate qualifications (Table 1). Demographics of non-respondents could not be obtained.

**Table 1:** Demographics, n (%)

	Overall (*n* = 368)	Service doctor (*n* = 101)	Basic trainee / Higher trainee (*n* = 108)	FM specialist (*n* = 156)
Gender				
Male	191 (52.6)	70 (69.3)	37 (34.6)	82 (53.6)
Female	172 (47.4)	31 (30.7)	70 (65.4)	71 (46.4)
Age				
25–34	102 (28.2)	11 (11.0)	80 (75.5)	11 (7.1)
35–44	201 (55.5)	36 (36.0)	25 (23.6)	140 (90.9)
45 or above	59 (16.3)	53 (53.0)	1 (0.9)	3 (1.9)
Cluster				
A	38 (10.4)	13 (12.9)	13 (12.0)	12 (7.7)
B	55 (15.0)	3 (3.0)	23 (21.3)	29 (18.7)
C	33 (9.0)	14 (13.9)	7 (6.5)	12 (7.7)
D	35 (9.5)	21 (20.8)	8 (7.4)	5 (3.2)
E	85 (23.2)	11 (10.9)	30 (27.8)	44 (28.4)
F	68 (18.5)	22 (21.8)	11 (10.2)	34 (21.9)
G	53 (14.4)	17 (16.8)	16 (14.8)	19 (12.3)
Place of graduation				
Hong Kong	312 (86.2)	68 (67.3)	101 (95.3)	143 (93.5)
Outside Hong Kong	50 (13.8)	33 (32.7)	5 (4.7)	10 (6.5)
Postgraduate qualifications (multiple answer)				
FHKAM (FM)^a^	170 (46.8)	6 (5.9)	12 (11.3)	152 (98.7)
FHKCFP^a^	210 (57.9)	23 (22.8)	47 (44.3)	139 (90.3)
FRACGP^a^	214 (59.0)	27 (26.7)	51 (48.1)	135 (87.7)
Diploma of FM	114 (31.4)	28 (27.7)	25 (23.6)	59 (38.3)
Membership/fellowship of other international colleges/board of FM/GP	26 (7.2)	9 (8.9)	9 (8.5)	8 (5.2)
Others	24 (6.5)	11 (10.9)	7 (6.5)	6 (3.8)

When the trainees finish basic training and passed conjoint examination, they are awarded FHKCFP/FRACGP. When the trainees pass higher training, they are awarded FHKAM (FM)

^a^*FHKAM (FM)* Fellow of the Hong Kong Academy of Medicine (Family Medicine), *FHKCFP* Fellow, Hong Kong College of Family Physicians, *FRACGP* The Fellowship of the Royal Australian College of General Practitioners

There was heterogeneity in teaching modalities and amount of training sessions for different types of GOPC doctors (Additional file 1: Table S1a) and across clusters (Additional file 1: Table S1b). While basic and higher trainees received most training, more than 80% of doctors received some form of training regardless of training status. Service doctors were more likely to have training via outside courses and SOPC attachments and higher trainees were more likely to receive research training. Cases, discussions and consultation skills feedback in terms of sit-in sessions and video reviews, were the most common training modalities (Table 3).

### Satisfaction of vocational training program

Eighty-two percent (82%) of participants were satisfied with their training. Around a quarter of respondents felt that they were not given a choice or enough time for their training and the training was not adequately broad or in depth (Table 2). Doctors who were dissatisfied with the training were more likely to also be dissatisfied with their clinical work (*p* < 0.001), their job as a medical doctor (*p* < 0.001), and their choice of being a family physician (*p* < 0.001). They were less likely to recommend working in GOPC to others and to students (*p* < 0.001) (Table 2). Doctors who attended external courses and sit-in consultations were more likely to be satisfied with FM training (Table 3).

**Table 2:** Satisfaction level and relationship with various attitudes; demographics

	Total	Satisfied (*n* = 295,81.9%)	Unsatisfied (*n* = 65,18.1%)	*P*-value^1^
Global Job Satisfaction	8.7 ± 1.5	9.1 ± 1.2	7.2 ± 1.6	< 0.001
Global Career Satisfaction	6.4 ± 1.0	6.6 ± 0.9	5.8 ± 1.2	< 0.001
Family Medicine (Global) Specialty Satisfaction	5.8 ± 1.1	6.0 ± 1.0	4.9 ± 1.3	< 0.001
Details of training				
I was given a choice as to what kind training I can receive				< 0.001
Agree	275 (75.5%)	253 (86.1%)	19 (29.2%)	
Disagree	89 (24.5%)	41 (13.9%)	46 (70.8%)	
I do not have an adequate number of training sessions over the last 12 months^a^				< 0.001
Agree	139 (38.8%)	89 (30.6%)	49 (76.6%)	
Disagree	219 (61.2%)	202 (69.4%)	15 (23.4%)	
I do not have protected time for training sessions^a^				< 0.001
Agree	88 (24.4%)	53 (18.1%)	33 (51.6%)	
Disagree	272 (75.6%)	240 (81.9%)	31 (48.4%)	
My training has prepared me to become a proficient doctor working in the GOPC				< 0.001
Agree	315 (87.0%)	279 (95.2%)	34 (52.3%)	
Disagree	47 (13.0%)	14 (4.8%)	31 (47.7%)	
My training was broad and in depth				< 0.001
Agree	270 (74.8%)	248 (84.9%)	19 (29.2%)	
Disagree	91 (25.2%)	44 (15.1%)	46 (70.8%)	
Likeliness to recommend the training received to others (1–10), mean ± sd	7.0 ± 1.7	1.8 ± 0.4	1.2 ± 0.4	< 0.001
0–6	106 (29.3%)	56 (19.0%)	48 (76.2%)	< 0.001
7–10	256 (70.7%)	239 (81.0%)	15 (23.8%)	
Demographics				
Cluster				0.018
A	38 (10.4%)	30 (10.2%)	8 (12.3%)	
B	55 (15.0%)	50 (17.0%)	4 (6.2%)	
C	33 (9.0%)	25 (8.5%)	8 (12.3%)	
D	35 (9.5%)	33 (11.2%)	1 (1.5%)	
E	85 (23.2%)	66 (22.4%)	18 (27.7%)	
F	68 (18.5%)	47 (16.0%)	18 (27.7%)	
G	53 (14.4%)	43 (14.6%)	8 (12.3%)	
Gender				0.453
Male	191 (52.6%)	156 (53.6%)	31 (48.4%)	
Female	172 (47.4%)	135 (46.4%)	33 (51.6%)	
Age				0.730
25–34	102 (28.2%)	85 (29.3%)	16 (25.0%)	
35–44	201 (55.5%)	157 (54.1%)	38 (59.4%)	
45 or above	59 (16.3%)	48 (16.6%)	10 (15.6%)	
Type of doctor				0.054
Service doctor	101 (27.7%)	74 (25.3%)	25 (38.5%)	
Basic trainee / Higher trainee	108 (29.6%)	93 (31.8%)	13 (20.0%)	
FM specialist	156 (42.7%)	125 (42.8%)	27 (41.5%)	
Place of graduation				0.489
Hong Kong	312 (86.2%)	249 (85.6%)	56 (88.9%)	
Outside Hong Kong	50 (13.8%)	42 (14.4%)	7 (11.1%)	

^1^Chi squared test

^a^ The questions were negative questions, in which the answer ‘agree’ would be a negative answer and ‘disagree’ would be a positive answer. Figures may not add up to total N due to missing data

**Table 3:** Comparison in satisfaction level of training for those who have attended different kinds of training modalities

	Attended			Not attended		*P*-value^2^
	N	median^a^ (IQR)	% of satisfied	N	% of satisfied	
Any modalities	309	6 (8.0)	85.4	46	58.7	< 0.001
External courses	141	4 (3.0)	87.9	214	78.0	0.018
Sit-in consultations	100	3 (5.8)	89.0	255	79.2	0.031
Case discussion	82	3 (13.0)	85.4	273	81.0	0.362
Video review	81	3 (3.0)	86.4	274	80.7	0.236
SOPC attachment	65	3 (2.0)	84.6	290	81.4	0.540
Practice management	64	2 (1.5)	90.6	291	80.1	0.047
Research related	46	2 (5.0)	84.8	309	81.6	0.595
TCM attachment	4	2 (0.8)	75.0	351	82.1	0.550

^2^ Chi squared test or Fisher Exact test

^a^ Median of number of modules attended

In the regression analysis model (Table 4), satisfaction level regarding training was associated with job satisfaction (OR 2.2; 95%CI: 1.5–3.2), likeliness to recommend the training to others (OR4.1; 95%CI: 1.7–10.3), autonomy to training modalities (OR 11.2; 95%CI: 4.6–27.1), been given protected time for training (OR 8.2;95%CI:3.2–21.4), and with training considered broad and in depth (OR 3.2; 95% CI: 1.3–7.9).

**Table 4:** Stepwise logistic regression model for satisfaction in regards to the training received in GOPC

	OR (95% CI)
Global Job Satisfaction	2.2 (1.5–3.2)
Likeliness to recommend the training received to others	
Score 0–6	1
Score 7–10	4.1 (1.7–10.3)
Details of training	
I was given a choice as to what kind of training I can receive	
Disagree	1
Agree	11.2 (4.6–27.1)
I do not have an adequate number of training sessions over the last 12 months^a^	
Agree	1
Disagree	8.2 (3.2–21.4)
My training was broad and in depth	
Disagree	1
Agree	3.2 (1.3–7.9)

^a^ The questions were negative questions, in which the answer ‘agree’ would be a negative answer and ‘disagree’ would be a positive answer

### Satisfaction with family medicine as a specialty career

Around 19% of participants would not want to work in their current job (GOPC) nor be a family physician given the choice, while 9% would rethink their career as a doctor given the chance [data not shown]. Older doctors (*p* = 0.023) and service doctors (*p* < 0.001) had significantly lower satisfaction levels. In the regression model, high job satisfaction (*p* < 0.001), autonomy to training modalities (*p* = 0.034), received training which is considered broad and in depth (*p* < 0.001) and have enrolled in FM training (*p* < 0.001) were associated with higher career satisfaction (Table 5).

**Table 5:** Association between family medicine (global specialty) career satisfaction score and various attitudes; demographics

	Bivariate association		Stepwise Multivariate regression (adj. *R*^2^ = 0.539)	
	Spearman’s r	*p*-value	Standardized Beta	*p*-value
Global Job Satisfaction	0.589	< 0.001	0.487	< 0.001
Global Career Satisfaction	0.405	< 0.001		
Details of training				
I am satisfied with the training provided in GOPC setting over the last 12 months	0.437	< 0.001		
I was given a choice as to what kind training I can receive	0.384	< 0.001	0.096	0.034
I do not have an adequate number of training sessions over the last 12 months^a^	0.150	0.004		
I do not have protected time for training sessions^a^	0.144	0.006		
My training has prepared me to become a proficient doctor working in the GOPC	0.486	< 0.001		
My training was broad and in depth	0.465	< 0.001	0.214	< 0.001
Likeliness to recommend the training received to others (1–10)	0.469	< 0.001		
	mean ± sd^c^	*p*-value^2^		
Demographics				
Cluster		0.029		
A	5.8 ± 1.2			
B	6.1 ± 1.0			
C^b^	5.2 ± 1.2			
D	5.8 ± 1.1			
E	5.9 ± 1.2			
F	5.8 ± 1.0			
G	6.0 ± 1.2			
Gender		0.125		
Male	5.7 ± 1.2			
Female	5.9 ± 1.1			
Age		0.023		
25–34	5.9 ± 1.0			
35–44	5.9 ± 1.2			
45 or above^b^	5.4 ± 1.2			
Type of doctor		< 0.001	0.181	< 0.001
Service doctor^b^	5.4 ± 1.2			
Basic trainee / Higher trainee	5.9 ± 1.1			
FM specialist	6.0 ± 1.1			
Place of graduation		0.357		
Hong Kong	5.9 ± 1.1			
Outside Hong Kong	5.7 ± 1.3			

^2^ANOVA or independent two samples t test

^a^scales inverted

^b^Significant different from other groups

^c^mean ± sd of Family Medicine (Global Specialty) Career Satisfaction Score

## Discussion

Overall satisfaction with the current FM training was high at 82% - a level of satisfaction comparable to or even higher than those reported in international studies. Although initial reports from the United Kingdom showed that around half of trainees were unsatisfied with their training in the early 1990s [1], subsequent efforts to improve the training [13] appears to have paid off, and the latest figures showed that more than 80–90% of doctors felt satisfied with their training and work [14–16]. Similarly, high levels of satisfaction with training, ranging from more than 80 to 90%, was found in a survey conducted in Ireland [17]. A more recent report from Dublin also found that only 1% of trainees indicated that they regretted training as a general practitioner [18]. By contrast, a survey conducted in the United States in 2008 found that only half of doctors who graduated within 10 years were satisfied with being a family physician and that satisfaction was related to the characteristics of the training program [4].

This study found that doctors who were given choices related to the training content and modalities, had higher training satisfaction. Future studies could explore if enhancing trainee’s autonomy to tailor the details of their training can enhance their satisfaction with their training and career. Meanwhile, doctors who were unsatisfied with their training were more likely to feel that they had an inadequate number of training sessions, that the training has not made them a proficient doctor working in GOPC, and be unsatisfied with their clinical work. As a weakness shared by all survey studies, it is not possible to determine if the doctors were unsatisfied with the training and therefore did not want to work in GOPC or whether the reverse was true: or that the doctors disliked working in GOPC and therefore were unsatisfied with the training, which was mostly conducted in GOPC. Lastly, this study also echoes previous studies that the training provided depends on location and individual trainers [9].

Furthermore, the current study agrees with the previous literature that good FM training programs are interlinked with good career satisfaction [4]. As with previous studies, this research reveals that FM doctors who considered their training as ‘broad and in-depth’, had higher career satisfaction [4]. In this study, service doctors were least satisfied with their career and many characteristics of training were associated with career satisfaction (Table 5). A worrisome finding is that around one-fifth of doctors regretted training as an FM doctor and around one-tenth would choose to rethink their career as a doctor, given the chance. We hypothesize that this could be due to the well-reported phenomenon of burnout in doctors [19, 20], but can only be determined in future studies.

As family medicine training programs are gaining in popularity [21], satisfaction surveys should be conducted as a part of a regular evaluation of the quality of training programs. As ‘no curriculum is perfect in design and delivery’, regular evaluation including satisfaction levels can help to ‘correct the deficiencies in a continuous and updated manner’ [22]. In fact, periodic satisfaction research or survey is conducted in countries such as the United Kingdom and Australia [14–16, 23, 24]. Satisfaction research can also be repeated after important changes are introduced in the training program to assess the impact of changes [24]. Specific instruments are being developed to measure satisfaction on different aspects of training in some countries [17, 22]. Future surveys may look further into satisfaction towards training in hospitals because there are discrepancies in the effects of hospital training on satisfaction [1, 14, 18]. Others unexplored factors that may be associated with satisfaction include: degree of feedback [17, 23] availability of hand-on experience [4, 23], psychological impact [25], and the use of logbooks [23].

### Strength and weakness

To the authors’ knowledge, this is the first quantitative study in primary care in Asia to describe the satisfaction of doctors towards their vocational training in family medicine. The sample size was adequate and satisfaction levels were found to be associated with a number of attitudes listed in Tables 2, 3 and 5. A baseline level on satisfaction level of family medicine training is now established and future surveys can be used to monitor and evaluate changes in satisfaction levels, especially after changes are implemented in the training curriculum. In addition, use of validated instruments enabled our exploration of satisfaction with training as well as satisfaction of family medicine specialty career.

Yet, the cross-sectional design of the current study did not allow us to draw causal conclusions and can be prone to recall bias. While the survey was anonymous, the location of GOPC was known as the survey was mailed back by internal mail. Participants may also report higher satisfaction with training or their FM career due to social desirability. Although the study achieved an acceptable response rate of 86.8%, we could not obtain non-respondents’ characteristics and; therefore, our findings may not be generalisable. In addition, limitations related to sample size contributed to wide confidence intervals and imprecise effect size estimation.

## Conclusion

The overall satisfaction level of physicians towards current family medicine training in Hong Kong is high. Having autonomy, protected time, external courses and individual feedback in sit in consultations s associated with higher training satisfaction levels. Finally having adequate breadth and depth of family medicine training is associated with family medicine career satisfaction.

## Supplementary information

**Additional file 1: Table S1.** a. Number of training sessions within protected time in the past 12 months by trainee status. b. Number of training sessions within protected time in the past 12 months by cluster (*n* = 363).

**Additional file 2: Appendix 1.** Selected items from the Physician Worklife Survey (PWS). **Appendix 2.** Finalized questionnaire.

## Data Availability

The datasets used and/or analyzed during the current study are available from the corresponding author on reasonable request.

## Abbreviations

FHKAM (FM): Fellow of the Hong Kong Academy of Medicine (Family Medicine)
FHKCFP: Fellow, Hong Kong College of Family Physicians
FM: Family medicine
FRACGP: The Fellowship of the Royal Australian College of General Practitioners
GOPCs: Government Primary Care Clinics
HA: Hospital Authority
HKCFP: Hong Kong College of Family Physician
PWS: Physician Worklife Survey
RACGP: Royal Australian College of General Practitioner
