# Supplementary material for: Family medicine vocational training and career satisfaction in Hong Kong
Source: BMC Fam Pract. 2019 Oct 20;20:139. doi: 10.1186/s12875-019-1030-8 (PMC6800987; doi:10.1186/s12875-019-1030-8)
Supplement: Supplementary file 1 — Additional file 1: Table S1. a. Number of training sessions within protected time in the past 12 months by trainee status. b. Number of training sessions within protected time in the past 12 months by cluster (n = 363). [file 12875_2019_1030_MOESM1_ESM.docx]

Table S1a. Number of training sessions within protected time in the past 12 months by trainee status

|  | Service doctor  (n=101) | Basic trainee / Higher trainee  (n=107) | Family medicine  Specialist (n=154) | p-value^1^ |
| --- | --- | --- | --- | --- |
| Any modalities* |  |  |  |  |
| N | 82 (81.2%) | 103 (96.3%) | 127 (82.5%) | 0.001 |
| median (IQR) | 4 (1.3) | 12 (30.0) | 4 (7.0) | <0.001 |
| Video review |  |  |  |  |
| N | 9 (8.9%) | 67 (62.6%) | 6 (3.9%) | <0.001 |
| median (IQR) | 3 (3.5) | 3 (4.0) | 3 (2.3) | 0.869 |
| Sit-in consultations |  |  |  |  |
| N | 19 (18.8%) | 72 (67.3%) | 9 (5.8%) | <0.001 |
| median (IQR) | 2 (1.0) | 4 (8.0) | 4 (5.0) | 0.155 |
| Case discussion |  |  |  |  |
| N | 14 (13.9%) | 61 (57.0%) | 8 (5.2%) | <0.001 |
| median (IQR) | 2 (2.5) | 4 (33.0) | 2 (12.3) | 0.148 |
| Practice management |  |  |  |  |
| N | 11 (10.9%) | 44 (41.1%) | 10 (6.5%) | <0.001 |
| median (IQR) | 2 (0) | 2 (1.8) | 3 (10.0) | 0.203 |
| Research related |  |  |  |  |
| N | 1 (1.0%) | 26 (24.3%) | 20 (13.0%) | <0.001 |
| median (IQR) | - | 1.5 (4.3) | 4 (4.8) | 0.193 |
| SOPC attachment |  |  |  |  |
| N | 31 (30.7%) | 18 (16.8%) | 15 (9.7%) | <0.001 |
| median (IQR) | 3 (1.0) | 14 (38.0) | 2 (2.0) | 0.001 |
| TCM attachment |  |  |  |  |
| N | 0 | 2 (1.9%) | 2 (1.3%) | - |
| median (IQR) | - | - | - | - |
| External courses |  |  |  |  |
| N | 35 (34.7%) | 28 (26.2%) | 81 (52.6%) | <0.001 |
| median (IQR) | 4 (2.0) | 2.5 (3.5) | 4 (3.0) | 0.015 |
| % of satisfied on training | 74.7% | 87.7% | 82.2% | 0.054 |

^1^ Kruskal Wallis tests were used for continuous variables, while chi-squared tests for categorical variables

* Sum of training session from different modalities may not add up to total number of training session for any modalities

Table S1b. Number of training sessions within protected time in the past 12 months by cluster (n=363)

|  | Cluster A  (n=38) | Cluster B  (n=54) | Cluster C  (n=33) | Cluster D (n=34) | Cluster E  (n=84) | Cluster F (n=66) | Cluster G  (n=53) | p-value^1^ |
| --- | --- | --- | --- | --- | --- | --- | --- | --- |
| Any modalities* |  |  |  |  |  |  |  |  |
| N | 34 (90%) | 45(83%) | 32(97%) | 34(100%) | 74(88%) | 56(85%) | 37(70%) | **0.001** |
| median (IQR) | 6 (6.3) | 10 (18.5) | 6 (5.0) | 4 (0) | 9.5 (16.0) | 4 (2.8) | 7 (18.5) | **0.006** |
| Video review |  |  |  |  |  |  |  |  |
| N | 11(29%) | 19(35%) | 2(6%) | 7(21%) | 25(30%) | 10(15%) | 8(15%) | **0.008** |
| median (IQR) | 3 (3.0) | 6 (11.0) | - | 2 (3.0) | 3 (4.0) | 3 (3.0) | 2 (2.0) | **0.037** |
| Sit-in consultations |  |  |  |  |  |  |  |  |
| N | 12(32%) | 13(24%) | 14(42%) | 7(21%) | 31(37%) | 9(14%) | 14(26%) | **0.019** |
| median (IQR) | 4 (3.5) | 2 (16.0) | 3 (26.0) | 2 (0) | 4 (10.0) | 2 (7.0) | 3 (2.0) | 0.155 |
| Case discussion |  |  |  |  |  |  |  |  |
| n | 10(26%) | 21(39%) | 12(36%) | 0(0%) | 18(21%) | 9(14%) | 13(25%) | **<0.001** |
| median (IQR) | 1.5 (4.8) | 6 (43.0) | 2.5 (8.0) | - | 3.5 (7.3) | 2 (4.0) | 12 (39.5) | **0.036** |
| Practice management |  |  |  |  |  |  |  |  |
| n | 6(16%) | 16(30%) | 4(12%) | 11(32%) | 17(20%) | 3(5%) | 8(15%) | **0.004** |
| median (IQR) | 2 (3.0) | 2 (10.0) | 2 (16.5) | 2 (1.0) | 2 (2.0) | 2 | 1 (0.8) | **0.031** |
| Research related |  |  |  |  |  |  |  |  |
| n | 4(11%) | 3(6%) | 5(15%) | 0 | 16(19%) | 8(12%) | 10(19%) | - |
| median (IQR) | - | 1 | 3 (5.5) | - | 4.5 (8.5) | 4.5 (7.0) | 2 (1.5) | **0.041** |
| SOPC attachment |  |  |  |  |  |  |  |  |
| n | 10(26%) | 4(7%) | 23(70%) | 5(15%) | 9(11%) | 3(5%) | 10(19%) | **<0.001** |
| median (IQR) | 7 (10.0) | 2 (21.8) | 3 (2.0) | 3 (2.0) | 1 (1.0) | 4 | 24 (36.3) | **<0.001** |
| TCM attachment |  |  |  |  |  |  |  |  |
| n | 0(0%) | 0(0%) | 1(3%) | 0(0%) | 3(4%) | 0(0%) | 0(0%) | - |
| median (IQR) | - | - | - | - | 2 | - | - | - |
| External courses |  |  |  |  |  |  |  |  |
| n | 16(42%) | 15(28%) | 9(27%) | 18(53%) | 36(43%) | 38(58%) | 12(23%) | **0.001** |
| median (IQR) | 4 (4.5) | 2 (3.0) | 3 (3.0) | 4 (0) | 3 (4.0) | 4 (1.0) | 8 (5.8) | 0.053 |
| % of satisfied on training | 78.9% | 92.6% | 75.8% | 97.1% | 78.6% | 72.3% | 84.3% | **0.018** |

^1^ Kruskal Wallis tests were used for continuous variables, while chi-squared tests for categorical variables

* Sum of training session from different modalities may not add up to total number of training session for any modalities
