# Supplementary material for: Family medicine vocational training and career satisfaction in Hong Kong
Source: BMC Fam Pract. 2019 Oct 20;20:139. doi: 10.1186/s12875-019-1030-8 (PMC6800987; doi:10.1186/s12875-019-1030-8)
Supplement: Supplementary file 2 — Additional file 2: Appendix 1. Selected items from the Physician Worklife Survey (PWS). Appendix 2. Finalized questionnaire. [file 12875_2019_1030_MOESM2_ESM.docx]

**Appendix**

Appendix 1. Selected items from the Physician Worklife Survey (PWS)

| **Global job satisfaction** |
| --- |
| I am satisfied with my clinical work in GOPC over the last 12 months |
| If I were to choose over again, I would not work in GOPC |
| I would recommend working in GOPC to others as a career |
| **Global career satisfaction** |
| All things considered, I am satisfied with my career as a doctor |
| If I were to choose over again, I would not be a doctor |
| **Global specialty (family medicine) satisfaction** |
| If I were to choose over again, I would choose to be a family physician |
| I would recommend FM to a student seeking advice |

Appendix 2. Finalized questionnaire

**Questionnaire on Training Opportunities in General Outpatient Clinics**

The Chinese University of Hong Kong has been appointed by the Food & Health Bureau in conjunction with HAHO to evaluate the Resources Allocation Exercise (RAE) Training Programme. In brief, **the RAE training program entitles each service doctor and higher trainee to have training within protected time** (which was previously not formally available).

This is an **ANONYMOUS QUESTIONNAIRE SURVEY**. All information will remain strictly confidential and will not be disclosed to any of your colleagues or supervisors.

Your responses to this questionnaire, which will ask questions about your training opportunities within GOPCs, will serve to improve the future training environment in GOPC.

Please return the completed questionnaire with the self-addressed envelope via internal mail to Mr. Lawrence Luk, School of Public Health, Prince of Wales Hospital, Shatin, NT or facsimile (2606-3500). Thank you for your cooperation.

**Section A: Details on training frequency and modalities**

| In the 12 months **from 1 April 2014 to 31 March 2015**: | | | |
| --- | --- | --- | --- |
| 1. | You have been working as a:  1. Service doctor □ 2. Higher trainee □ 3. Basic trainee □ 4. Family Medicine specialist □  (please tick one) | | |
| 2. | How many training sessions within protected time were allocated to you?  (Protected time means having training within working hours purely reserved for the purpose of training) | __________ sessions | |
| 3. | What modalities of training were they?  Please indicate the number of sessions as best as you can. | Video review: | ____ sessions |
|  |  | Sit-in consultations: | ____ sessions |
|  |  | Case discussion: | ____ sessions |
|  |  | Practice management: | ____ sessions |
|  |  | Research related: | ____ sessions |
|  |  | SOPC attachment: | ____ sessions |
|  |  | TCM attachment: | ____ sessions |
|  |  | Outside courses: | ____ sessions |
|  |  | Details of outside courses: | |

**Section B: Satisfaction towards GOPC & training**

|  | **Strongly disagree** | **Disagree** | **Agree** | **Strongly agree** |
| --- | --- | --- | --- | --- |
| 1. I am satisfied with my clinical work in GOPC over the last 12 months | □ | □ | □ | □ |
| 1. I am satisfied with the training provided in GOPC setting over the last 12 months | □ | □ | □ | □ |
| 1. All things considered, I am satisfied with my career as a doctor | □ | □ | □ | □ |
| 1. If I were to choose over again, I would not work in GOPC | □ | □ | □ | □ |
| 1. If I were to choose over again, I would not be a doctor | □ | □ | □ | □ |
| 1. I would recommend working in GOPC to others as a career | □ | □ | □ | □ |
| 1. If I were to choose over again, I would choose to be a family physician | □ | □ | □ | □ |
| 1. I would recommend FM to a student seeking advice | □ | □ | □ | □ |
| 1. I was given a choice as to what kind training I can receive | □ | □ | □ | □ |
| 1. I do not have an adequate number of training sessions over the last 12 months | □ | □ | □ | □ |
| 1. I do not have protected time for training sessions | □ | □ | □ | □ |
| 1. My training has prepared me to become a proficient doctor working in the GOPC | □ | □ | □ | □ |
| 1. My training was broad and in depth | □ | □ | □ | □ |

1. All things considered, how likely are you to recommend the training you received to others?

| Very unlikely | | | |  | |  | |  | | Extremely likely | | | |
| --- | --- | --- | --- | --- | --- | --- | --- | --- | --- | --- | --- | --- | --- |
| 1 | 2 | 3 | 4 | | 5 | | 6 | | 7 | | 8 | 9 | 10 |
| □ | □ | □ | □ | | □ | | □ | | □ | | □ | □ | □ |

15. Comments/ Suggestions for improving the RAE training programme:

|  |
| --- |

| **Section C: Your Personal Details:**  **Please tick or enter the appropriate information as appropriate** | | |
| --- | --- | --- |
| 1. | Which HA cluster did you work in most of the time during 1 April, 2014 to 31 March, 2015?  1. HKE □ 2. HKW □ 3. KC □ 4. KE □ 5. KW □ 6. NTE □ 7. NTW □ | |
| 2. | Age:  25-29 □ 30-34 □ 35-39 □ 40-44 □ 45-49 □ 50-54 □ 55-59 □ 60 or above□ | |
| 3. | Gender: M □ F □ | |
| 4. | Year of obtaining basic medical degree (e.g. 1998, 2003): ___________ | |
| 5. | Place of graduation: Hong Kong □ Outside Hong Kong □ | |
| 6. | Postgraduate qualifications: FHKAM (FM)  FHKCFP  FRACGP  DFM  Membership/fellowship of other international colleges/board of FM/GP  Others:_____________________________ | yes □ no □  yes □ no □  yes □ no □  yes □ no □  yes □ no □ |

**- END OF SURVEY -**
